# Ultra-compact multi-task processor based on in-memory optical computing

**DOI:** 10.1038/s41377-025-01814-0

**Published:** 2025-03-24

**Authors:** Wencan Liu, Yuyao Huang, Run Sun, Tingzhao Fu, Sigang Yang, Hongwei Chen

**Affiliations:** 1 Department of Electronic Engineering, Tsinghua University, Beijing, China; 2 Beijing National Research Center for Information Science and Technology (BNRist), Beijing, China; 3 Hunan Provincial Key Laboratory of Novel Nano Optoelectronic Information Materials and Devices, College of Advanced Interdisciplinary Studies, National University of Defense Technology, Changsha, China

**Keywords:** Integrated optics, Photonic devices

## Abstract

To enhance the computational density and energy efficiency of on-chip neuromorphic hardware, this study introduces a novel network architecture for multi-task processing with in-memory optical computing. On-chip optical neural networks are celebrated for their capability to transduce a substantial volume of parameters into optical form while conducting passive computing, yet they encounter challenges in scalability and multitasking. Leveraging the principles of transfer learning, this approach involves embedding the majority of parameters into fixed optical components and a minority into adjustable electrical components. Furthermore, with deep regression algorithm in modeling physical propagation process, a compact optical neural network achieve to handle diverse tasks. In this work, two ultra-compact in-memory diffraction-based chips with integration of more than 60,000 parameters/mm^2^ were fabricated, employing deep neural network model and the hard parameter sharing algorithm, to perform multifaceted classification and regression tasks, respectively. The experimental results demonstrate that these chips achieve accuracies comparable to those of electrical networks while significantly reducing the power-intensive digital computation by 90%. Our work heralds strong potential for advancing in-memory optical computing frameworks and next generation of artificial intelligence platforms.

## Introduction

The swift progression in artificial intelligence has catalyzed significant advancements across a multitude of domains, including computer vision^1,2^, natural language processing^3,4^, and autonomous driving^5^, primarily through the deployment of deep neural networks (DNNs)^6–8^. Nevertheless, the reliance on conventional electronic platforms^9–11^, such as central processing units (CPUs) and graphics processing units (GPUs), for complex computations often leads to escalated power consumption and demands for more extensive computational resources. These traditional electronic architectures, largely anchored in the von Neumann computing model which distinguishes between memory and processing units, incur considerable energy and time expenditures during the data exchange process, a limitation particularly pronounced in DNN-intensive computing environments^12,13^. Conversely, optical neural networks (ONNs), which harness the interactions between light and optical devices, can execute computing processes in a singular step, thereby circumventing the need for data transmission. This inherent characteristic of ONNs underscores their growing recognition for energy efficiency, expansive bandwidth, and the potential for parallel processing at the speed of light^14–17^.

In recent times, on-chip ONNs have emerged as promising alternatives to their electronic counterparts, achieving performance on par with cutting-edge digital processors through innovative approaches such as interference techniques employing Mach–Zehnder interferometers^18–22^, wavelength multiplexing with microring modulator arrays^23–27^, material advances of utilizing the phase change material as trainable optical neurons^28–30^, optimization of metasurface for optical computing in various applications^31–34^ and diffraction methods utilizing sub-wavelength units^35–41^. These on-chip ONN architectures not only match the computational prowess of state-of-the-art processors but also offer superior computing speed and density while consuming less energy, thanks to the efficiency of light-speed computation, garnering increasing attention from the research community. Interference and time-wavelength multiplexing methods boast high reconfigurability, attributed to the adjustable nature of each component, theoretically enabling them to accommodate a variety of tasks. Nevertheless, these approaches face challenges in scaling due to the physical footprint of each unit and the necessity for continuous energy supply, limiting their potential for large-scale expansion. Recent advancements of the diffraction-based method, known as the on-chip diffractive optical neural network (DONN), were inspired from free space DONN^42–46^, which is based on discrete diffractive units with a relatively large volume. On-chip DONN adopts different modeling and design rules with integrated one-dimensional dielectric metasurfaces comprised of entirely passive sub-wavelength units that function as trainable parameters. The CMOS-compatible process enables on-chip DONN to offer advantages of large-scale manufacturing, device miniaturization, and the elimination of additional calibration processes compared with free space DONN. This method excels in encoding a vast number of parameters and connections into optical parameters, showcasing exceptional computational capabilities. However, the passive characteristic of the computational units implies that once fabricated, the sub-wavelength units lack adjustability, presenting a drawback in terms of reconfigurability and multitasking capability. Ref. ^47^ proposed a free space diffractive optical neural network structure based on transfer learning, but still limited to theoretical simulation.

In this study, we introduce an innovative learning architecture for DONN utilizing a silicon on insulator (SOI) platform. Drawing inspiration from transfer learning theory and employing the hard parameter sharing method^48–50^, our approach involves mapping the majority of trained parameters in neural networks onto the DONN chip. As a result, these parameters are fixed, and the chip functions as a shared optical core (SOC). Significantly, the SOC chips facilitate in-memory computing using optical methods, thereby obviating the need for the traditional memory access process inherent in the von Neumann architecture. Consequently, owing to the diffraction-based architecture, the in-memory computing operations within SOC chips are both rapid and highly energy-efficient. These characteristics render them particularly advantageous for extensive computing tasks in compact, low-power devices such as edge devices. Subsequently, a lightweight network, comprising a minimal number of parameters, is serially connected to the DONN chip, forming a lightweight transfer network (LTN). This configuration enables the system to perform multiple tasks by interchanging different LTNs, while integrating most parameters and computations onto optical diffraction chips for data preprocessing. This can then be coupled with lightweight networks at different terminals for specific tasks, harnessing the benefits of low power consumption and extensive neural mapping capabilities of diffraction chips, while eschewing direct end-to-end training to preserve transferability and reconfigurability. Unlike traditional diffractive networks^35,37,39,51^ that act solely as classifiers or encoders, the SOC can simultaneously offer the benefits of optical feature extraction and data compression, while overcoming the non-reconfigurable limitations of previous designs, making it more suitable for practical applications. To validate our network architecture, we fabricated two transferable SOC chips, conducting empirical tests for classification and regression tasks. For classification, we utilized three distinct datasets, employing the SOC+LTN configuration to achieve an average accuracy of 95.9%. This accuracy matches that of three large-scale electrical networks while reducing the digital computation parameters by ~90%. In the regression task, we leveraged the SOC as a multi-kernel convolution unit via a structure reparameterization algorithm, demonstrating its transferability to various classic convolution kernels through LTNs. The convolutional performance was tested on the Modified National Institute of Standards and Technology (MNIST) dataset for handwritten digit recognition, forming a CNN with SOC+LTN and electrical fully connected (FC) layers, achieving an accuracy of 95.0%. Our work lays a foundational stone for the synergy of future in-memory optical computing architectures and heralds strong potential for advancing optical computing frameworks and the next generation of artificial intelligence platforms.

## Results

### Principle

**Fig. 1:**
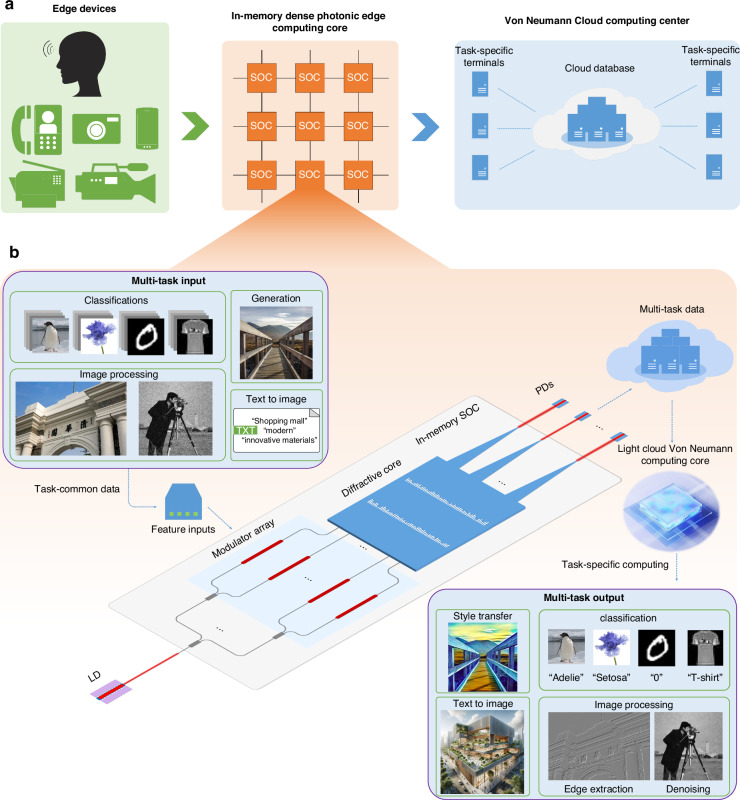
Schematic illustration of diffractive multi-task processing system and edge applications. **a** Schematic diagram of the proposed system in edge computing. The in-memory dense photonic computing core in edge devices process multi-task inputs. The processed data will be saved and conduct further task-specific computing in cloud. **b** Schematic diagram of the overall system processing. LD laser diode, SOC shared optical core, PD photodetector. Features processed from multi-task input are modulated into the SOC in phase form. The output results from PDs are saved in database as multi-task data, and eventually processed by task-specific light cloud Von Neumann computing core

In neural networks, computations are generally accomplished through two components: the multiplication of different input data with the same trained weights. This principle applies to both fully connected networks and convolutional neural networks. In conventional electronic processors, this process requires simultaneous access to both the input data and the corresponding weights from memory. In the optical in-memory computing approach in this work, the trained neural network weights are integrated into the chip as sub-wavelength diffractive elements. This means the network itself is stored as sub-wavelength silica slots of varying lengths, effectively mapping the computational parameters onto a specific physical structure on the chip. Consequently, during computation, it is only necessary to modulate the input data, eliminating the need to access network weights from memory. The architecture of the proposed multi-task system for edge computing is depicted in Fig. 1a, b, highlighting its capacity to handle diverse computational tasks. This versatility is achieved through the innovative design of the SOC, which is not task-specific but rather designed to work in conjunction with various LTNs to tackle different challenges. As illustrated in Fig. 1a, upon acquiring information, the edge device processes multi-task inputs through a series of internal SOCs, which enable high-speed and low-power data handling. Subsequently, the processed data are transmitted to the cloud database (DB). Depending on the specific task requirements, diverse processing tasks are executed on various terminals. Specifically, the process begins with the input data obtained by edge devices, which undergo feature preprocessing and is introduced to the system. This data modulation is accomplished with a modulator array, allowing the necessary input information to be encoded onto the incident light and then channeled into the SOC chip via waveguides. Within the SOC, there are two hidden layers comprising subwavelength silica (SiO_2_) slots. These slots with fixed width and thickness, and length acts as the trainable parameter. Owing to the integration of these parameters into diffraction units, the units possess both storage and computational capabilities, thus making the SOC process become passive in-memory computing. After passing through the SOC, the chip’s output is captured by photodetectors (PDs), marking the completion of the optical processing phase. This output can then be transmitted and stored in cloud DB, and interfaced with different LTNs across various terminals, facilitating a comprehensive processing flow. This system’s flexibility is a significant departure from traditional integrated DONN chips, which are typically engineered to perform a singular, fixed task. In contrast, the SOC’s functionality is augmented by the subsequent electrical processing stage, where specific LTNs undertake final task-specific processing. Data from various sources are encoded onto the light in phase form and processed through the in-memory SOC, engaging in a dense, all-optical preprocessing phase that encompasses feature recognition and extraction. The extracted features are then conveyed through three waveguides, normalized, and stored for further processing. These preprocessed results are finally fed into specific LTNs responsible for the final task execution, showcasing the system’s adaptability and efficiency in handling diverse computational tasks. This unique approach underscores the potential of integrating optical and electrical components to achieve high performance in artificial intelligence applications.

The training of the entire network system includes two parts, namely SOC training and LTN training. However, during the training process, these two parts are carried out simultaneously, that is, SOC needs to be combined with different LTNs and jointly calculate the loss of multiple tasks. For classification tasks, the loss function is shown in Eq. (1):

1 $$Loss=-\mathop{\sum }\limits_{t=1}^{T}\left[\frac{1}{{N}_{t}}\sum _{i}\mathop{\sum }\limits_{c=1}^{{M}_{t}}{y}_{ic}\log ({p}_{ic})\right]$$

where *T* is the number of training tasks, *N*_*t*_ is the number of samples in the *t* dataset, *M*_*t*_ is the number of categories. *y*_*ic*_ is a sign function (0 or 1), if the true category of sample *i* is equal to *c*, take 1; otherwise, take 0, and *p*_*ic*_ is the prediction probability of sample *i* belonging to category *c*.

For regression tasks, the loss function can be calculated as Eq. (2):

2 $$Loss=\mathop{\sum }\limits_{t=1}^{T}\left[\frac{1}{{N}_{t}}\mathop{\sum }\limits_{i=1}^{n}{(\widehat{{y}_{i}}-{y}_{i})}^{2}\right]$$

where *T* is the number of training tasks, *N*_*t*_ is the number of samples in the *t* dataset. $\widehat{{y}_{i}}$ is the predicted result, and *y*_*i*_ is the ground truth data.

**Fig. 2:**
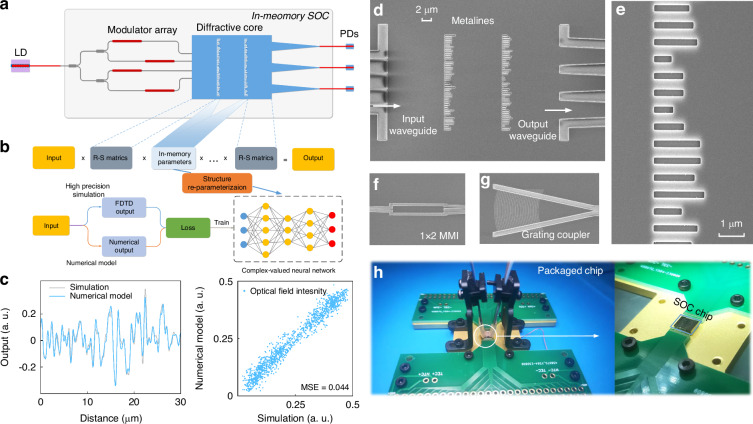
Numerical modeling and packaged SOC chip. **a** The on-chip structure and the modeling diagram of the designed SOC. **b** Numerical modeling based on structure re-parameterization algorithm, with Rayleigh-Sommerfeld (R-S) matrics representing the propagation between adjacent layers and a complex-valued neural network representing the propagation in metaline. **c** Comparison of intensity in the output plane of optical field calculated by the proposed numerical model and finite-difference time-domain (FDTD) simulation. **d**–**g** SEM images of SOC chip, SiO_2_ slots, 1 × 2 MMI splitter, and grating coupler. **h** Photograph of the optically and electrically packaged SOC chip

The optimization of the SOC and LTNs revolves around minimizing the loss function to determine the optimal parameters. To achieve a high level of integration and computational density in the SOC, while concurrently reducing energy consumption and improving model accuracy, we employed a deep regression neural network (DRNN) during the training phase as a surrogate for the conventional modeling of light propagation through the hidden layers^40,52^. This approach is illustrated in Fig. 2a, b where modeling between adjacent layers is governed by the Rayleigh-Sommerfeld diffraction model, but within the hidden layers, the propagation is substituted by the DRNN with complex-valued parameters through a process known as structural reparameterization^53,54^. The DRNN, trained on massive Lumerical finite-difference time-domain (FDTD) data, is capable of achieving accuracy comparable to that of the FDTD simulation. This substitution notably shortens the optimization process time while maintain considerable accuracy, as depicted in Fig. 2c and Fig. S1a, b. Furthermore, the DRNN sidesteps the restrictive approximation conditions inherent to traditional DONN designs, which often necessitate additional layer spacing and multiple slots to simulate a single parameter (further elaborated in Supplementary note 1). This advancement markedly enhances the integration density and lowers the computational energy requirements. Upon substituting the conventional DONN model with a DRNN-based model amenable to gradient optimization, the entire system can be globally optimized through a back-propagation algorithm. Following the completion of this parameter optimization phase, the SOC can be fabricated for use in various tasks, with the LTNs tailor-made for specific applications. This methodology not only streamlines the process of optical neural network design but also significantly elevates the efficiency and versatility of the resulting computational architecture.

In this study, we embarked on a multifaceted validation of our proposed system architecture through both classification and regression tasks, employing specifically SOC chips. These chips, featuring two hidden layers, each comprising 50 subwavelength SiO_2_ slots and equipped with four input and three output ports, were crafted to assess the network’s performance across different computational tasks. For the classification analysis, we leveraged three distinct datasets: the Iris, Wheat Seed, and Palmer Archipelago (Antarctica) Penguin datasets, to train the SOC and LTNs. This training process involved a collaborative optimization of the network parameters using all three datasets. The optimization utilized weighted cross-entropy functions, as specified in Eq. (1), to optimize the same SOC and three divergent LTNs. Subsequent to normalizing the SOC chip’s output optical power, the LTNs were tasked with deciphering the precise classification outcomes based on their outputs. For the regression analysis, a separate SOC chip was utilized. Our recent research has validated the capability of DONN output ports to facilitate convolution result^51^. Nevertheless, given that the kernel is inherently static, the same chip was restricted to executing only a fixed convolution kernel. To address this limitation and enhance the system’s reconfigurability, we implemented a training approach that allowed for the DONN’s reconstruction via LTNs. This method enabled the three output channels of the SOC to process varied multi-kernel convolution results simultaneously through the LTNs. During the convolution operations, a two-dimensional (2D) image was initially segmented into a 2 × 2 window matrix, aligned with the SOC’s input dimensions. This window sequentially navigated through the image in both horizontal and vertical directions, employing a stride of one. The resultant 2 × 2 matrices were then transformed into one-dimensional (1D) vectors and modulated onto the optical field through phase modulators for input into the SOC. For instance, a 28 × 28 image was condensed into 729 (27 × 27) vectors for SOC input, with its output optical power normalized before being fed into the LTNs to derive three sets of convolved vector outputs (3 × 729). These outputs were subsequently reorganized into a 27 × 27 2D matrix to finalize the results of the three sets of convolutions (3 × 27 × 27).

We fabricated the SOC chips to perform the classification and convolution tasks with two layers on an SOI wafer, the scanning electron microscope image as shown in Fig. 2d. In diffractive core, two successive subwavelength silica metalines with gap of 15 μm modulate the diffracted light’s phase in slab waveguide. A grating coupler array with 127 μm pitch is utilized for the fan-in and fan-out between SOC chip and fiber array (FA) with coupling loss of 5 dB/facet at a wavelength of 1550 nm, and then two stages of 1 × 2 multimode interferometer (MMI) trees split the coupled laser equally across four parallel input silicon waveguides. Four waveguides with TiN heaters are employed as phase modulators for phase modulation. Figure 2e–g depicts the SiO_2_ slots, MMI, and grating coupler structures. Figure 2h shows the photograph of an optically and electrically packaged SOC chip, employing the electron beam lithography (EBL) process. Optical signal fans in and out via a vertically coupled FA, while the tensor data loading operates via a double-layer bonded d.c. wire, following the electrical current paradigm.

### Experimental results of classification

**Fig. 3:**
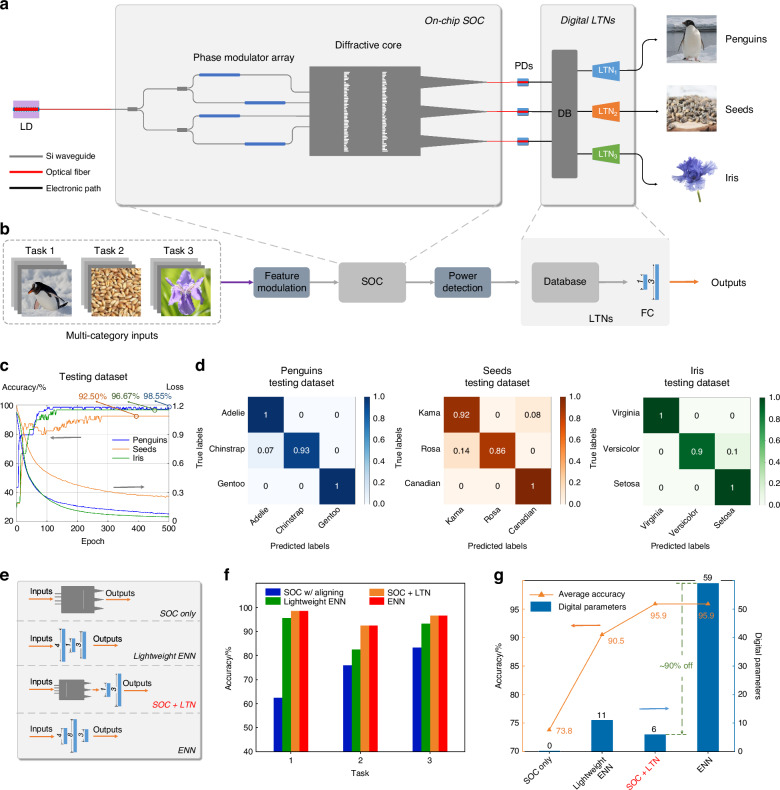
Schematic illustration and experimental results for muti-category classification task. **a** Schematic diagram of the overall system for classification tasks. DB database, LTN lightweight transfer network. **b** Experimental configuration of input and process flow of the system. FC fully connected network. **c** Accuracy and loss trajectories of the system throughout the training process. **d** Experimental confusion matrics of three testing datasets. **e** Four distinct network architectures in comparison. ENN electrical neural network. **f** Accuracies of the four networks in three tasks. **g** Comparisons of the number of digital parameters and average accuracies of the four networks

To verify the multitasking proficiency of the proposed optical computing system, we undertake classification experiments using three distinct datasets: the Palmer Penguin Classification Dataset, the Wheat Seed Classification Dataset, and the Iris Flower Classification Dataset. Figure 3a, b delineates the comprehensive processing workflow of the system, where the SOC, serving as a preprocessing unit, is equipped to handle the aforementioned classification tasks. This unit, boasting a substantial parameter count, initiates the signal loading procedure, whereby characteristic signals are converted into phase change of the incident light at 1550 nm with phase modulation. Following this modulation, the light signal traverses the diffraction structure, passing through two layers of pre-trained hidden layers (metalines), and then detected by three PDs. The normalized output optical power is stored in a data aggregation center, which constitutes the preliminary output results. These results are then conveyed to the subsequent LTNs for further processing. The digital component of the LTNs comprises three distinct lightweight terminal networks, each tailored to one of the classification tasks. Notably, these FC networks, incorporating two layers with one and three neurons respectively, embody a total of six digital parameters, rendering them significantly lightweight in comparison to the SOC’s 100 parameters and thereby substantially mitigating the computational demand on the terminal electrical network. Figure 3c exhibits the accuracy and loss trajectories of the system throughout the pre-training phase across the three datasets, illustrating the system’s capacity to achieve high task-specific accuracies. An essential aspect of the experimentation involved a singular, fixed calibration and compensation operation on the SOC aimed at diminishing the diffraction structures’ sensitivity to minute variations in manufacturing precision and procedural inaccuracies, with subsequent fine-tuning of the FC networks. The alignment of the system’s accuracy with the simulated outcomes is exemplified, and the confusion matrix is presented in Fig. 3d. The empirical classification accuracies obtained for the Penguin, Wheat Seed, and Iris datasets were 98.55%, 92.50%, and 96.67%, respectively, underscoring the system’s robust classification capabilities across diverse tasks.

Building on the aforementioned experiment, to highlight the efficiency of the SOC, we conducted performance evaluations on the three datasets using four distinct network architectures, as illustrated in Fig. 3e. These architectures include the compensated SOC only, a lightweight electrical neural network (ENN), our proposed SOC+LTN system, and a larger parameter scale ENN. The scale and parameter composition of these networks vary significantly, with the SOC representing the DONN described in prior research, characterized by its purely optical operations and absence of digital parameters. However, its fixed structure limits its multitasking capabilities, resulting in generally lower recognition performance across the three tasks, with an average accuracy of 73.8%. To underscore the utility of SOC, we employed three lightweight ENNs, each comprising 11 parameters, to directly classify the datasets, achieving an average accuracy of 90.5%, as shown in Fig. S3. Yet, the implementation of our proposed SOC+LTN structure, which reduces the count of digital parameters in each lightweight electrical network to only 6, markedly improved the average accuracy to 95.9%. This performance matches that of larger ENNs containing 56 digital parameters each while realizing ~90% savings in electrical computation, as shown in Fig. S4. This significant reduction indicates that the majority of computations are efficiently conducted via all-optical processes within the SOC. Detailed comparisons of classification results and parameter efficiencies are presented in Fig. 3f, g. These results demonstrate that by leveraging the hard parameter-sharing method inherent in transfer learning, our system can match the classification performance of electrical networks with significantly larger parameter scales. It accomplishes this by predominantly utilizing optical operations, thereby not only conserving computational resources but also enhancing multitasking capabilities. This underscores the proposed system’s capability to integrate optical computing elements with transfer learning strategies to achieve high efficiency and versatility in task processing. The detailed comparison of the above four structures is presented in Table S3.

### Experimental results of regression

**Fig. 4:**
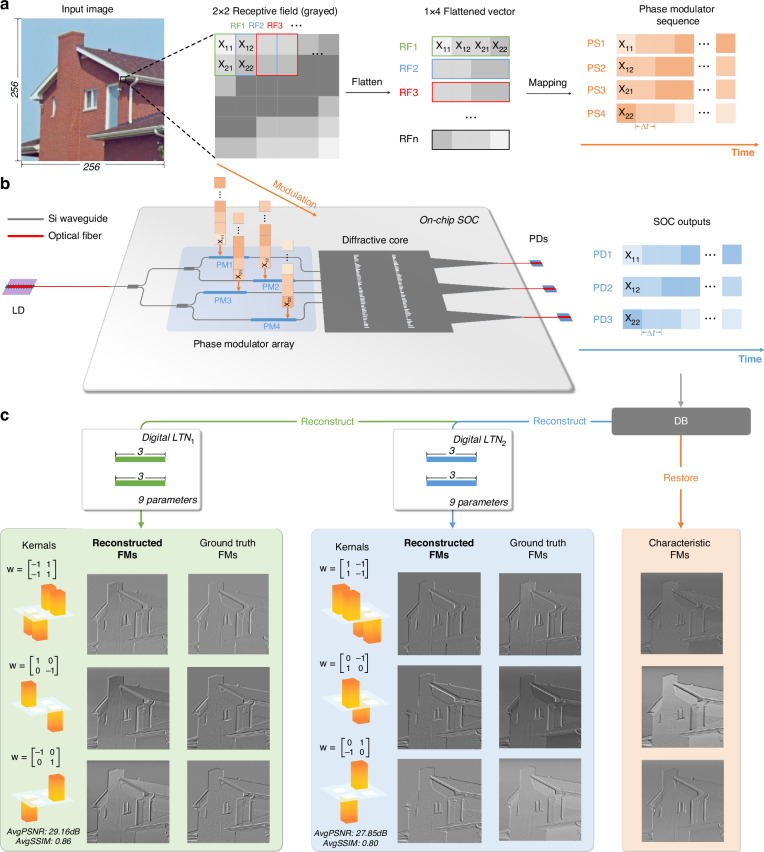
Experimental principles and reconstruction results of SOC in multi-kernel regression task. **a** Pre-processing and mapping of image input data for SOC. **b** Data modulation process and overall concept in regression tasks of SOC chip. **c** Experimental outputs of specific reconstructed convolution kernels and the characteristic kernels of the SOC, compared with the ground truth from the commercial 64-bit computer. FM feature map

To validate the capability of the proposed system for regression tasks, we deploy neural network regression algorithms to configure the system as an image preprocessing unit, endowed with the capability to execute distinct convolutional kernel functions. With this method, the advanced SOC presented in this study is utilized to facilitate multi-kernel convolution operations and can be dynamically reconfigured through LTN to accommodate multi-kernel transfer, thereby fulfilling the requirements of various specific convolutional kernels. Illustrated in Fig. 4a, b, the fundamental processing principles of the system are outlined. Initially, input images are converted to grayscale and segmented into 2 × 2 pixel blocks, correlating with the dimensions of the convolution kernel. Each block is then linearized into a 1 × 4 pixel strip, which is subsequently mapped onto a plane comprising spatial channels and temporal sequences, generating a restructured pixel sequence that aligns with the chip’s four inputs. The interval preceding adjacent pixels denotes the system’s modulation period, Δt, facilitating the modulation of input image data onto the phase of the incoming light in accordance with convolutional operation. This modulated signal then traverses through the diffraction structure’s two hidden layers, culminating in the parallel temporal output of processing results at varied positions on the output plane. The trio of output sets can be directly amalgamated to derive three distinct sets of convolutionally processed results, referred to as the feature outputs of the SOC. Moreover, with the aid of LTNs, these output sets can further undergo reconstruction with lightweight FC networks, thus facilitating the generation of convolutional feature maps for additional, designed multi-kernel convolutions. Notably, this reconstruction process can simultaneously generate three designed new convolutional kernels, substantially enhancing the versatility and reconfigurability of on-chip diffraction structures. The experimental results, as delineated in Fig. 4c, Fig. S5, S6, underscore the effectiveness of the proposed system in applying convolution operations on images. This includes both the outcomes of reconstructed convolution kernels and the characteristic outputs of the SOC. To rigorously assess the adaptability of the system, six canonical fixed convolution kernels were chosen as benchmarks for transferring. These kernels were categorized into two sets, with two FC networks deployed to reconstruct the outputs derived from the SOC. The comparative analysis of the output feature maps from the system against those obtained from purely electronic convolutional processes revealed the average peak signal-to-noise ratio of 29.16 dB and 27.85 dB, alongside the average structural similarity index measure of 0.86 and 0.80, respectively. In this task, a total of 9 2 × 2 convolution operations were completed with SOC+LTN. The digital parameters of SOC+LTN network and electronic network are 18 and 36, respectively. Therefore, the in-memory computing architecture reduces the power-intensive digital computation in regression task by around 50%. These metrics attest to the high degree of similarity between the two outputs, confirming the system’s capability to reconstruct and fulfill its designated functions.

**Fig. 5:**
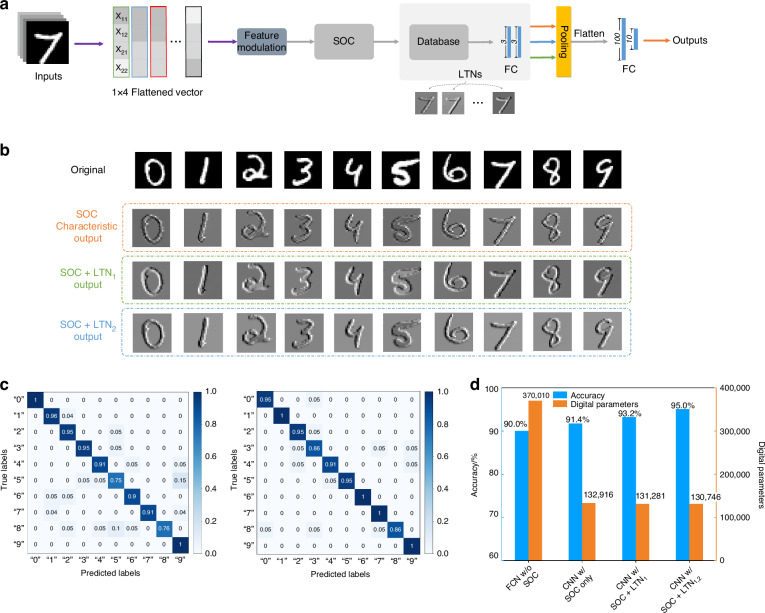
Experimental configuration and results of multi-kernel regression system in MNIST dataset classification. **a** Experimental configuration of CNN for MNIST handwritten digit recognition based on SOC and LTNs. **b** Partial experimental results of MNIST handwritten digits feature maps produced by SOC, SOC + LTN_1_ and SOC + LTN_2_, respectively. **c** Confusion matrics of classifications conducted solely through SOC and combined with LTN_1_ and LTN_2_. **d** Comparison of accuracies and digital parameters in four distinct network architectures

Further validation of the system’s reconstructive efficiency was undertaken through image classification experiments on the MNIST handwritten digit recognition dataset, which is also based on the algorithm of multi-kernel regression. The procedural workflow, illustrated in Fig. 5a, initiates with the input image being linearized and mapped according to Fig. 4a, and subsequently fed into the SOC chip. The ensuing results are normalized and relayed to the LTNs to generate a variety of input image feature maps, as delineated in Fig. 5b and Fig. S7. This is followed by a 4 × 4 pooling process with a stride of 1, after which the data undergoes flattening and classification via an FC network, comprising 100 and 10 neurons in each layer, with softmax serving as the nonlinear activation function to deliver the ultimate classification outcome. To benchmark the system’s performance, comparisons were drawn against four distinct network models: an electrical FC network, a convolutional neural network (CNN) with SOC feature extraction, a CNN with SOC feature extraction and an LTN, and a CNN with SOC feature extraction complemented by two LTNs. Figure 5c presents the confusion matrices for classifications conducted solely through SOC and combined with LTN_1_ and LTN_2_, respectively. As depicted in Fig. 5d, the classification accuracy and parameter count for the four systems highlight a progressive enhancement in classification accuracy without a corresponding increase in the overall number of network parameters. The training curve of the above four structures is shown in Fig. S8. Notably, the experimental classification accuracy for the test set of the proposed system reached 95.0%, rivaling that of same-scale electrical convolutional neural networks. This outcome accentuates the reconfigurability of the proposed preprocessing system, showcasing its potential to streamline computational operations while maintaining performance efficiency.

## Discussion

This study has employed chips with 53 μm in length and 30 μm in width, which integrate 100 parameters each. Thus, the SOC chips achieve an integration density of 100/(53 μm × 30 μm) = 62,893 parameters/mm^2^, significantly surpassing previous on-chip optical computing structures. Additionally, the adoption of FDTD, structure reparameterization methods, and deep regression neural networks for modeling marked a substantial improvement in modeling accuracy over approximate numerical modeling methods. As a result, due to the significant increase in computing unit density, the unit area computing density of the SOC chip in this work has also been greatly improved and the chip’s insertion loss in our experiments has been reduced by over 12 dB compared with our previous works^35,51^, with the overall insertion loss ~8.5 dB (further elaborated in Supplementary note 2). Operating at a modulation speed of 10 kbaud and comprising two layers with 50 designed parameters each, the SOC’s active computational throughput can be calculated as [3 × (4 × 50^2^ − 50) + 2 × (6 × 50)] × 10^4^ = 0.30 GOPS as noted in refs. ^45,55^. The chip area of SOC is 30 μm × 53 μm = 1.59 × 10^−3 ^mm^2^, thus the computational density is about 0.30/(1.59 × 10^−3^) = 0.19 TOPS/mm^2^, which is an order of magnitude higher than previous work^35–38^. The primary limitation lies in the relatively low modulation rate of the thermal optical modulation scheme employed. Assuming the integration of plasma dispersion effect-based silicon modulators in our design with a moderate 10 Gbaud modulation speed, the calculated density can be increased to 191 POPS/mm^2^.

However, due to the nature of linear operations, this approach may not be entirely accurate when viewed from the perspective of linear computations. If the nonlinearity at the output detection is disregarded, and the internal computation of the SOC chip is treated as a complex linear operation, then the size of the linear operation kernel is 4 × 3. This means each computation involves 4 × 3 + 3 × 3 = 21 OPS. Therefore, at a modulation speed of 10 Gbaud, the computational capacity is calculated as 21 × 10^10^/(1.59 × 10^−3^) = 132.1 TOPS/mm^2^. However, we believe that when optimizing parameters for an unknown target task, determining computational density based solely on input-output dimensions is also not entirely fair. As shown in Fig. S10, the performance clearly improves as the number of computational units increases, even though the number of input and output ports remains unchanged. This suggests that increasing the number of internal computational units, while keeping the number of ports constant, can enhance the system’s computational capacity. Since we do not initially know the exact form of the computation needed, the only feasible approach is to use larger parameters and computational density for implicit representation and processing tailored to specific tasks. We believe that both of these computational schemes (fully connected computation and input-output linear equivalence) do not adequately represent the computational capacity of the diffractive optical computing chip. This issue warrants further in-depth study and exploration in future research.

Due to the overall power consumption of the SOC chip including external devices being 271.51 W (the modulation power of the chip is only 0.01 W), the energy efficiency of SOC can be calculated as 191 × 10^3^/271.51 = 703.5 TOPS/(W⋅mm^2^) with the first computing method mentioned above. Notably, the power consumption of external devices can be further reduced by using lighter devices or dedicated chips. The Energy efficiency of the SOC chip is further elaborated in Supplementary note 3. Table 1 shows the comparison between SOC chips and other on-chip optical computing architectures, as well as mainstream electronic computing architectures. The stability of the experimental system and fabrication errors of the SOC chip are elaborated in Supplementary note 8.

**Table 1:** Comparison between SOC and other on-chip computing architectures

Source	Technology	Unit size	Integration in theory (parameters/mm^2^)	Computational density (TOPS/mm^2^)	If reconfigurable	Energy efficiency (/mm^2^)
Shen et al.^18^	MZI	55 μm × 220 μm	10	N/A	Yes	N/A
Feldmann et al.^28^	WDM + PCM	127 μm × 127 μm	60	162	Yes	0.065 TOPS/W
Ashtiani et al.^25^	MRM	N/A	N/A	1.75	Yes	8 TOPS/W
Fu et al.^35^	Diffraction	1.5 μm × 2 μm	~2000	4600^a^	No	16.94 TOP/W
Wang et al.^37^	Diffraction	1 μm × 2.5 μm	~6000	3 × 10^4^^a^	No	N/A
Xu et al.^55^	Diffraction + MZI	N/A	150	1758	Yes	6.75 TOPS/W
NVIDIA A100 PCIe^58^	MOSFET	N/A	N/A	0.66	Yes	1.7 × 10^−3^ TOPS/W
NVIDIA H100 PCIe^59^	MOSFET	N/A	N/A	1.71	Yes	2.4 × 10^−3^ TOPS/W
Our work	SOC + LTN	0.5 μm × 3 μm	>60,000	1.91 × 10^5^^a^	Yes	703.5 TOPS/W

^a^Calculated under 10 Gbaud modulation speed

In the multitasking scheme proposed in this study, the SOC chips with in-memory computing obviate the need for the traditional memory access process inherent in the von Neumann architecture. The computing process of SOC chips only requires loading the data without loading the weights that need to be multiplied or added. Thus, based on diffraction units, the in-memory computing operations within SOC chips render them particularly advantageous for extensive computing tasks in compact, low-power devices with rapid and highly energy-efficient processing. The lightweight supporting network used is based on electrical network implementation, which will also become a factor limiting the overall system energy consumption and working bandwidth. We believe that the supporting lightweight network can also be replaced by other adjustable optical computing architectures, greatly improving system bandwidth and achieving all optical transferable learning system. This scheme can combine the advantages of optical diffraction chips with a large number of parameters, as well as the adjustable advantages of other on-chip optical computing approach. The work of this study can provide a foundation for the complementarity of future optical computing architectures.

In conclusion, our research introduces a novel design methodology that enhances the integration and reconfigurability of in-memory optical diffraction chips. Through the application of the hard parameter sharing framework derived from transfer learning principles, this approach enables the fixation of the majority of parameters on ultra-compact, in-memory, yet non-adjustable optical diffraction chips, facilitating multitasking in tandem with lightweight transfer networks. Through a series of classification tasks and convolutional regression tasks, we have demonstrated the multitasking capability of chips designed using this method. In terms of classification tasks, employing the SOC+LTN architecture, we managed to process the bulk of parameters and computational complexity on the optical chip, boasting low power consumption and high bandwidth, and achieving performance comparable to electronic computing architectures while reducing electronic computational complexity by 90%. In terms of regression tasks, our recent work has shown that the SOC can independently complete multi-core convolution tasks, although its convolution kernels are fixed post-manufacture. However, with the utilization of LTN, it becomes possible to reconfigure the SOC to perform other specific convolution kernel multitasking operations effectively. Furthermore, applying the SOC+LTN architecture to the MNIST handwritten digit recognition task led to an enhanced classification accuracy of 95.0% without increasing the number of parameters, underscoring the system’s efficiency. Finally, this work proposes an advancing in-memory optical computing framework, which provides a potential for future large-scale high-performance computing systems.

## Materials and methods

### Modeling and fabrication of SOC chip

On the basis of the DRNN above, the on-chip electromagnetic propagation can be modeled with DRNN and Huygens Fresnel principle, which is modified according to the restricted propagation conditions^40^, as depicted in Fig. S9. The propagation process of optical field between adjacent layers of SOC can be described as shown in Eq. (3):

3 $$a(m,n)=\frac{1}{j\lambda }\cdot \left(\frac{1+\cos \theta }{2r}\right)\cdot \exp \left(j\frac{2\pi r{n}_{slab}}{\lambda }\right)\cdot \gamma \exp (j{{\Delta }}\phi )$$

where *m* represents the point on the chip located at (*x*_*m*_, *y*_*m*_), *n* represents the point on the chip located at (*x*_*n*_, *y*_*n*_), *λ* is the working wavelength, *n*_*slab*_ is the effective refractive index (ERI) of the slab waveguide, $\cos \theta =({x}_{n}-{x}_{m})/r$, $r=\sqrt{{({x}_{n}-{x}_{m})}^{2}+{({y}_{n}-{y}_{m})}^{2}}$ is the distance between point *m* and point *n* on the chip, *γ* is a specific coefficient of the amplitude and Δ*ϕ* is a fixed phase delay for the classical Huygens-Fresnel principle when light propagates a certain distance in a slab waveguide.

Utilizing the DRNN for propagation within hidden layers and Eq. (3) for propagation between every two successive layers allows for a comprehensive description of the light. Due to the accuracy of the modeling result compared with the FDTD simulation result, without compromising accuracy, consider any *x* plane in the given SOC as the secondary source of the wave vector (*n* dimensions) based on the simulation accuracy. Therefore, for two *x* planes at a fixed distance, the propagation process can be articulated as a propagation matrix *A* (*n* × *n*). The elements within this matrix can be represented by Eq. (3), signifying the optical field propagated from the *i*-th pixel in the *x*_1_ plane to the *j*-th pixel in the *x*_2_ plane, which can be expressed as Eq. (4):

4 $$\left\{\begin{array}{c}{E}_{y}({x}_{2})={E}_{y}({x}_{1})\cdot A({x}_{2}-{x}_{1})\\ {A}_{i,j}=a(i,j)\\ {E}_{y}({x}_{3})=F[{E}_{y}({x}_{2}),{L}_{slot}]\end{array}\right.$$

where *E*_*y*_(*x*) is a row vector representing the electric field of plane *x*, *A* is the propagation matrix with the correction factors for the distance from *x*_1_ to *x*_2_, and *a*(*i*, *j*) can be expressed as Eq. (3). *F* is the function generated by the DRNN, *L*_*slot*_ refers to the length of the slots in the metaline. The matrix *A* and the propagation in each metaline can be calculated by Eq. (4), both of which represent the overall on-chip propagation process.

The output inverse taper functions as a mode selector to filter the optical field at the output facet of SOC to be single mode, for which we use eigenmode theory to describe^56^. According to the theory, any optical field with the electrical and magnetic components of *E* and *H* can be expanded into superposition of their eigenmode components *E*_*n*_ and *H*_*n*_ as expressed as Eq. (5) and (6), with a coefficient of an and bn respectively, which can be addressed by the overlapping integrals as shown in Eqs. (7) and (8):

5 $$E=\mathop{\sum }\limits_{n=0}^{N}{a}_{n}\cdot {E}_{n}$$

6 $$H=\mathop{\sum }\limits_{n=0}^{N}{b}_{n}\cdot {H}_{n}$$

7 $${a}_{n}=\frac{{\int}_{S}E\times {H}_{n}^{* }\cdot dS}{{\int}_{S}{E}_{n}\times {H}_{n}^{* }\cdot dS}$$

8 $${b}_{n}=\frac{{\int}_{S}{E}_{n}^{* }\times H\cdot dS}{{\int}_{S}{E}_{n}\times {H}_{n}^{* }\cdot dS}$$

Consequently, the inverse tapers select the fundamental mode component E_0_ and H_0_ of the optical filed only and drop other high-order modes away. The total taper-filtered power of the *i*-th output port can be calculated as *P*_*i*_ = *a*_0_ × *b*_0_.

The SOC chip was fabricated on an SOI platform with a 220 nm top layer silicon (Si) and a 2 μm dioxide layer via the process of EBL. The subwavelength metalines were fabricated by etching 220 nm Si film and a 2 μm thick silica upper cladding was deposited on the Si film. Thin layer of titanium nitride (TiN) was deposited as heaters with an average resistance of 281.4 Ω, and metal wire of AlCu (Cu:0.5%) was patterned as the the electrical connection to the electrodes and heaters.

### Training and numerical simulation

The modeling and training process of SOC and LTNs were conducted on five Nvidia 3090 GPUs with Pytorch 1.8.0, Cuda 11.2, and Keras 2.0 deep learning frameworks. The optical field of the trained SOC was numerically validated and the training data for DRNN was collected by Lumerical Variational FDTD. The modeling and training process of DRNN were conducted with complexPyTorch^57^, which can be obtained from https://github.com/wavefrontshaping/complexPyTorch.

### Experimental setup

A tunable laser diode (Santec TSL-550) was utilized with tuned power and wavelength of 13 dBm and 1550 nm respectively, and a polarization controller (PC) was used to ensure the optical signal is TE- 337 polarized. Single mode FA with 127 μm pitch was grating-coupled to the SOC chip for the optical fan-in and fan-out. A 16-bit muti-channel programmable current source (SiliconExtream MCVS6400-A) was employed for the data loading. A programmable multi-channel optical power meter (CommPolar C2500-OPM16) with a minimum power limit of −75 dBm was utilized for the receiving of the SOC outputs. A PC with an intel core i7-11800H CPU is utilized as the database and terminals with LTNs. A homemade control penalty based on Python script was built for the data loading, receiving, and synchronizing.

## Supplementary information

Supplementary information for Ultra-compact multi-task processor based on in-memory optical computing

## Data Availability

The data that support the findings of this study are available from the corresponding author upon reasonable request.
