# Supplementary material for: Ultra-compact multi-task processor based on in-memory optical computing
Source: Light Sci Appl. 2025 Mar 24;14:134. doi: 10.1038/s41377-025-01814-0 (PMC11930997; doi:10.1038/s41377-025-01814-0)
Supplement: Supplementary file 1 — Supplementary information for Ultra-compact multi-task processor based on in-memory optical computing [file 41377_2025_1814_MOESM1_ESM.pdf]

Supplementary information for

Ultra-compact multi-task processor based on in-memory optical computing

Wencan Liu[1,2], Yuyao Huang[1,2], Run Sun[1,2], Tingzhao Fu[3], Sigang Yang[1,2], and Hongwei Chen[1,2,*]

[1]Department of Electronic Engineering, Tsinghua University, Beijing 100084, China

[2]Beijing National Research Center for Information Science and Technology (BNRist)

[3]Hunan Provincial Key Laboratory of Novel Nano Optoelectronic Information Materials and Devices, College of Advanced Interdisciplinary Studies, National University of Defense Technology, Changsha 410073, China

[*]Correspondence: chenhw@tsinghua.edu.cn

1 Supplementary note 1: Deep regression neural network setup

In our previous studies [1–3], the process of neuron mapping was typically reduced to the phase alteration of the optical field produced by the length variations of silica slots. However, the relationship between the optical field and these slots is governed by a complex function that is challenging to articulate in a direct mathematical expression, therefore we use slots group to represent one single neuron. We also increase the layer spacing to mitigate the impact of the angle of incident light, thereby stabilizing the effective refractive index (ERI) and facilitating a more precise mathematical analysis. In the present research, we employ the DRNN empowered by ComplexPyTorch [4–6] to approximate the intricate function representing light propagation within a line of slots, referred as metaline [7]. Assuming that the width, thickness and lattice constant of the slots are fixed, and their length is arranged as L_{slot}. The maximum length of each slot is L. The starting position of a metaline is x_0, and the electrical field distribution of the optical field at x0 is E_{x_0}, so the field distribution of the optical field at $(x_0 + L)$ µm can be calculated according to Eq. (S1):

$$E_{x_0+L} = F(E_{x_0}, L_{slot}) \tag{1}$$

where F is the complex function of light propagating in the metaline, and when the width, thickness and lattice constant of the slot are fixed, the optical field succeeding the metaline $E_{x_0} + L$ is only related to the optical field preceding the metaline and the length of the slots. Therefore, it is only necessary to find the mapping function F, giving the length of the slots in the metaline and the optical field preceding the metaline, then the optical field succeeding the metaline can be quickly calculated. With this method, the neuron mapping process can be represented by neural network, which no longer requires slot group and can take effect at any layer spacing. In pursuit of the mapping function F, the approach involves using the deep regression neural network (DRNN) to approximate the light propagation process within the metaline. For this work, we employ 2.5D FDTD to generate both training and testing datasets.We utilize the ComplexPyTorch library [41,42] within the Pytorch platform to build the model's architecture due to its capability to handle complex-valued data.Assuming that the data size is N, in the training process, complex-valued mean square error (CMSE) is used as the loss function, which is defined as Eq. (S2):

$$CMSE(x, y) = \frac{1}{N}\sum_{N}\left[(x_{real} - y_{real})^2 + (x_{imag} - y_{imag})^2\right] \tag{2}$$

where x is the output of DMRM and y is the ground truth of var-FDTD simulation. Since x and y are both complex-valued, xreal and ximag are the real and imaginary part of x, and the same for y_{real} and y_{imag}. Goodness of fit (R^2) is chosen as the measure of fitting effect of the DMRM, which can be calculated as Eq. (S3):

$$R^2 = \frac{ESS}{TSS} = 1 - \frac{RSS}{TSS} = \frac{1}{N}\sum_{N}\frac{\sum_{i=1}^{n}(\widehat{y_i} - \overline{y_i})^2}{\sum_{i=1}^{n}(y_i - \overline{y_i})^2} \tag{3}$$

where y_i is the ground truth, $\widehat{y_i}$ is the DMRM output and $\overline{y_i}$ is the Average value of y_i. Here consider both the real part and the imaginary part of the predicted optical field and ground truth.

With this method, to validate the effectiveness of the trained DRNN, the simulational results for w/ diffraction units and w/o diffraction units are shown as Fig. S1. In the training process, the loss curves of training set and verification set are shown in Fig. S2(a). The goodness of fit (R^2) curves of the validation

set is shown in Fig. S2(b). Fig. S2(c) illustrates that the post-training DRNN prediction results closely align with the simulation results. This high degree of similarity, especially evident in the overall trend, peaks, and valleys of the waveform, provides substantial evidence of the effectiveness of the DRNN.

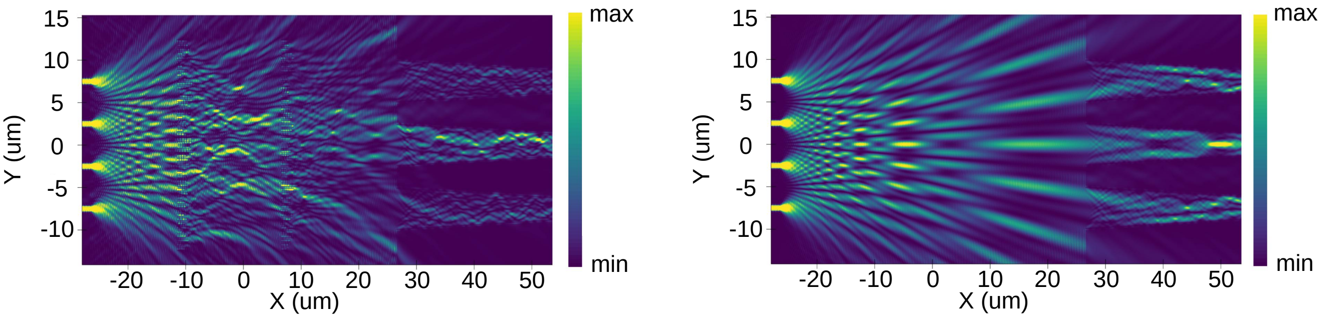

Figure S1: Simulational propagation results for w/ diffraction units (left) and w/o diffraction units (right), highlight the modulation ability of the SOC in an ultra-compact structure.

2 Supplementary note 2: Insertion loss and experimental errors

In our experiment, the operating wavelength of the incident laser was 1550 nm, and the input power was measured to be 5 dBm. The total power detected at the three output ports of SOC was approximately -16.5 dBm, indicating an overall insertion loss of approximately 21.5 dB. In order to measure the loss of the designed SOC chip, we divide the insertion loss into two parts: coupling loss between fibers and the chip and waveguide loss, and the loss of the diffraction core of SOC chip. For this purpose, we designed a contrast structure on the chip, including the same coupling structure and waveguide design, and the length of the waveguide is also approximately the same. After measurement, the loss of this control structure is approximately 13 dB, which means the total loss is 13 dB when the incident light does not pass through the diffraction core of SOC chip. Combining the overall insertion loss of 22.5 dB, it can be concluded that the insertion loss in the diffraction core is around 8.5 dB. In our previous works, due to the utilization of approximate modeling methods, the overall size of the chip is relatively large, and the measured insertion loss in the diffraction core reached more than 20 dB. Compared to our previous method, the proposed modeling method not only improves the chip integration, but also reduces the loss of diffraction core by more than 12 dB. Thus, smaller size of the chip means higher detection power values, thereby reducing the impact of noise, which also demonstrates the superiority of high integration chips and the proposed method. The comparison of insertion loss of the proposed SOC and previous on-chip DONN is shown in Table. S1.

We also found that there are errors in insertion loss and experimental results between the fabricated SOC chip and the simulational design. We believe that the errors may come from the signal loading, chip fabrication, and signal detection stages. By utilizing more advanced processing equipment, the errors introduced during chip fabrication can be fundamentally reduced. Additionally, incorporating perturbations into the data input during network training can further enhance the network's robustness. Finally, more advanced network training methods will also improve the error tolerance of the SOC chips.

Table S1: **Insertion loss of the proposed SOC and previous on-chip DONN.**

diffraction-based chip	distance between adjacent layers	Insertion loss
DONN [1]	300 um	33.2 dB
OCU [8]	300 um	21.4 dB
C-DONN [7]	15 um	6.9 dB (simulation)
**SOC**	**15 um**	**8.5 dB**

3 Supplementary note 3: Computational energy efficiency

The power consumption of SOC chip mainly comes from five parts: external laser source, on-chip thermal phase shifters, external current source, photodetectors and digital backend. We conclude the power consumption of SOC chip and LTNs in Table. S2 as listed in the follow:

Table S2: **Estimated power consumption of SOC + LTN system.**

Components	Power (W)
External laser source (benchtop)	100
External current source (benchtop)	120
Digital controller and LTNs (powered by intel core i7-11800H)	50
On-chip thermal phase shifters	0.01
Photodetectors	1.5
Total power consumption	271.51

Note that the average resistance of the utilized TiN heaters was measured as 281.4 Ω. Consequently, for the on-chip thermal phase shifters, each with an average tuning current of 3 mA, were employed, generating a power consumption of 0.01 W. Furthermore, 3 photodetectors, each with a power consumption of 0.5 W, were utilized, resulting in an overall power consumption of around 1.5 W. The digital processor was responsible for feature mapping and launching LTNs, with an estimated power consumption of 30 W, using an Intel core i7-11800H CPU. Consequently, the total power, including benchtop instruments such as an external laser source with a power consumption of 100 W and an external current source with a power consumption of 120 W, can be calculated as 271.51 W. As the calculated computational throughput of the SOC at 10 Gbaud modulation speed is 304 TOPS and 191 POPS·mm^{-2}, The energy efficiency of the SOC chip can then be calculated as 1.12 TOPS·W^{-1} and 703.5 TOPS·W^{-1}·mm^{-2}. The energy efficiency of SOC is not high due to the peripheral power consumption, which will not obviously increase as the scale of SOC increases.

The in-memory computation proposed involves integrating the computing weights directly into the chip using silica slots, allowing them to interact directly with the input light when data is loaded. The computing process of SOC chips only requires loading the data without loading the weights that need to be multiplied or added. Therefore, once the signal is loaded during the computing process (phase modulation is used in this work), the subsequent computing process is power-consumption free, which is significant. The digital parameters of SOC+LTN network and electronic network in multi-dataset classification task is 6 and 59, respectively. The digital parameters of SOC+LTN network and electronic network in regression task is 18 and 36, respectively. Therefore, the in-memory computing architecture reduces the power-intensive digital computation in classification and regression tasks of this work by around 90% and 50%, respectively. Although the energy consumption of signal loading and external devices supporting the signal loading process in this work is relatively high, we believe that the energy consumption can be solved through better fabrication methods and more specialized and lightweight external loading devices. The passivity of SOC chip computing process is significant.

4 Supplementary note 4: Parameter settings of SOC

The proposed SOC chips with 4 inputs and 3 outputs and two layers each containing 50 subwavelength silica slots are designed for the following reasons: First, the inputs and outputs are designed aiming to certain tasks. The 4 inputs and 3 outputs correspond to three multi-kernel convolutions with a 2×2 kernel size for the regression task discussed in regression tasks. The input and output dimensions are adequate for the multi-task scenarios examined in classification tasks. Second, the 2 hidden layers, each containing 50 subwavelength silica slots, were optimized for the task. Our analysis indicated that increasing the number of layers and neurons does not significantly enhance accuracy, as validated in Fig. S10.

5 Supplementary note 5: Scalability of SOC

In this work, we have significantly enhanced the scalability of on-chip DONN, innovatively enabling the same chip to adapt to different tasks. In this section, we will discuss the scalability challenges that SOC chips encounter when addressing more complex tasks.

The limitation of input/output dimensions has been a persistent challenge for on-chip optical computing systems. While it is relatively straightforward to expand the input-output dimensions and the number of slots, hidden layers within a certain range [1], scaling to higher dimensions poses significant challenges, particularly regarding size and insertion loss. However, several solutions to improve the scalability of SOC are available, such as parallel multi-chip processing and the use of one-hot encoding for limited outputs, which can map to a larger variety of output types [9] (e.g., 8-channel one-hot encoding can generate 256 output types). Although these methods may lead to a slight reduction in accuracy, they significantly increase the number of output channels and are applicable to our system.

6 Supplementary note 6: Operating wavelength of SOC

Our chip design approach involves first using computer simulations, tailored to the intended application scenario, to determine the specific parameters of its computational units. Then we proceed with fab-

rication, producing a chip capable of delivering the required computational functionality. Because the operational wavelength influences the computational function of the chip's structural units, it is essential to select the appropriate operating wavelength during the simulation stage. This chosen wavelength is subsequently applied in the experimental phase.

To ensure that our network training and structural design processes are effective, we first established the operating wavelength before fabrication. Based on this predetermined wavelength, we utilized Eq. (3) and (4) in the manuscript to simulate the on-chip optical propagation and computation. In this study, we selected 1550 nm, a standard communication wavelength, as our system's operating wavelength from the outset of our design and simulation. So, in the experimental process after chip fabrication, we choose the 1550 nm wavelength that matches the design process.

After fabrication, we also experimentally performed a wavelength-scan of the two SOC chips, the results of which are presented in Fig. S11, which indicates that at the initially chosen wavelength of 1550 nm, the system achieved performance consistent with simulation while maintain lower loss. Significant deviations in wavelength can lead to substantial discrepancies between the chip's computational performance and its intended functionality, while also causing a marked increase in overall insertion loss.

7 Supplementary note 7: Fabrication process of SOC

The SOC chip was fabricated on an SOI platform with a 220nm top layer silicon (Si) and a 2 µm dioxide layer via the process of EBL. The subwavelength metalines were fabricated by etching 220nm Si film and a 2 µm thick silica upper cladding was deposited on the Si film. In the optimized configuration, each metaline, measuring 30 µm in width (W), is perceived as a hidden layer and distributed along the Y-axis. As depicted in Fig. S12, the length and width of a metaline are set at 3 µm and 30 µm respectively. Each slot is characterized by a thickness (n) of 220 nm and a width (m) of 200 nm. The slots are spaced 300 nm apart (q), and the maximum length of each slot (p) is equal to the metaline's width (i.e., 3 µm). Fig. S12 (a) shows the detailed fabrication parameters and the cross sectional images of the SOC chip we fabricated. Fig. S12 (b) shows some key parameters of our designed diffractive core of SOC. The length of the slab (Si) waveguide is 53 µm and the width is 30 µm. The thickness of each slot is n = 220 nm, the width is m = 200 nm, the max length is p = 3 um, and the distance between adjacent slots is q = 300 nm.

8 Supplementary note 8: Stability and fabrication errors of SOC

Regarding the stability and errors of the chip and overall system during the experiment, we categorize the errors into two types: instability-related errors during experimental system testing and system errors introduced by chip fabrication and manufacturing processes. Concerning the stability of the experimental system, our testing system demonstrates relatively high stability, and thanks to the structural design, our thermal modulation operates reliably without the need for additional TEC device. To verify the reliability of the SOC chip and the experimental system, we measured the output results of the SOC chip with the same set of data under two different time with reconnection of the experimental system and compared the two, as shown in Fig. S13. The results indicate that the system maintains good stability and is not affected by fluctuations of the testing environment. Concerning the fabrication error of the SOC chip, we

acknowledge that fabrication errors like the EBL patterned lines may lead to slight structural variations in different chips. However, during the simulation process, our parameter optimization algorithm is based on gradient descent, which endows the optimized model with a certain degree of generalization ability and robustness against noise. Additionally, our team has conducted multiple fabrications of on-chip diffractive optical neural networks within the same facility, and through several iterations of technology and accumulated manufacturing experience, we have minimized errors induced by the fabrication process. During the experiments, we observed that the impact of inter-chip variations on the final results was not significant.

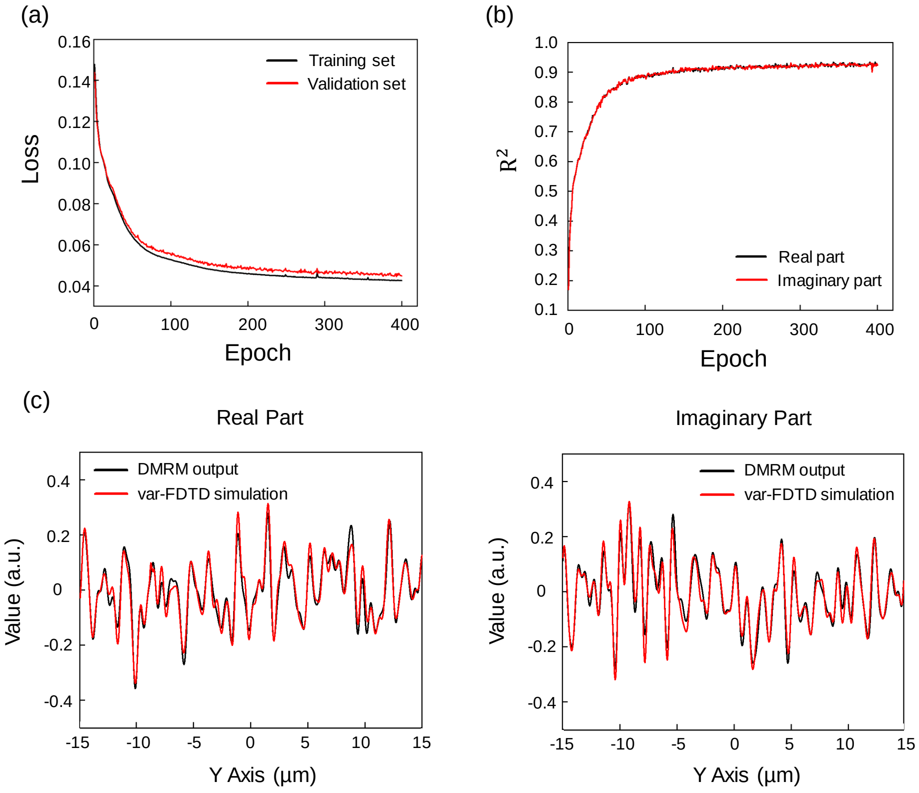

Figure S2: (a) The loss curves on the training set (black line) and validation set (red line) for the DMRM during the fitting procedure. (b) The goodness of fit (R^2) curves on the real part (black line) and imaginary part (red line) of the validation set during the learning procedure. (c) The comparison between the DRNN output and var-FDTD simulation results after the training process for the same sample in testing set.

Table S3: **comparison between four network structures**

structure	test accuracy of seed dataset	test accuracy of penguins dataset	test accuracy of iris dataset	number of digital parameters
SOC w/ aligning	75.9%	62.3%	83.3%	0
Light electrical network	82.5%	95.6%	93.3%	11
electrical network	92.5%	98.6%	96.7%	59
**SOC + LTN**	**92.5%**	**98.6%**	**96.7%**	**6**

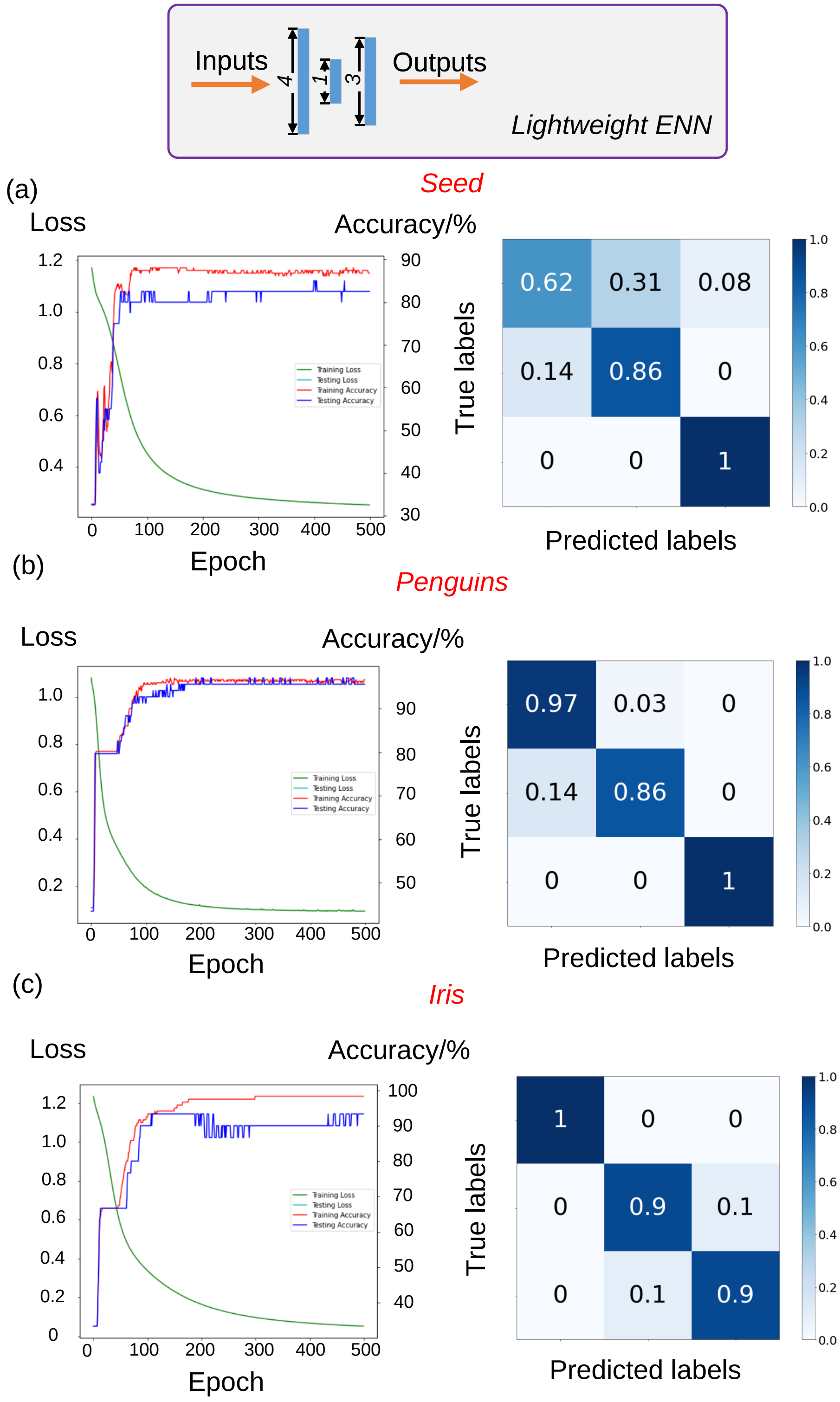

Figure S3: The accuracy and loss curves and confusion matrics of testing dataset for Light electrical net structure in (a) seed, (b) penguins, (c)iris dataset, respectively. The test accuracies for the 3 datasets are 82.5%, 95.6% and 93.3%, respectively.

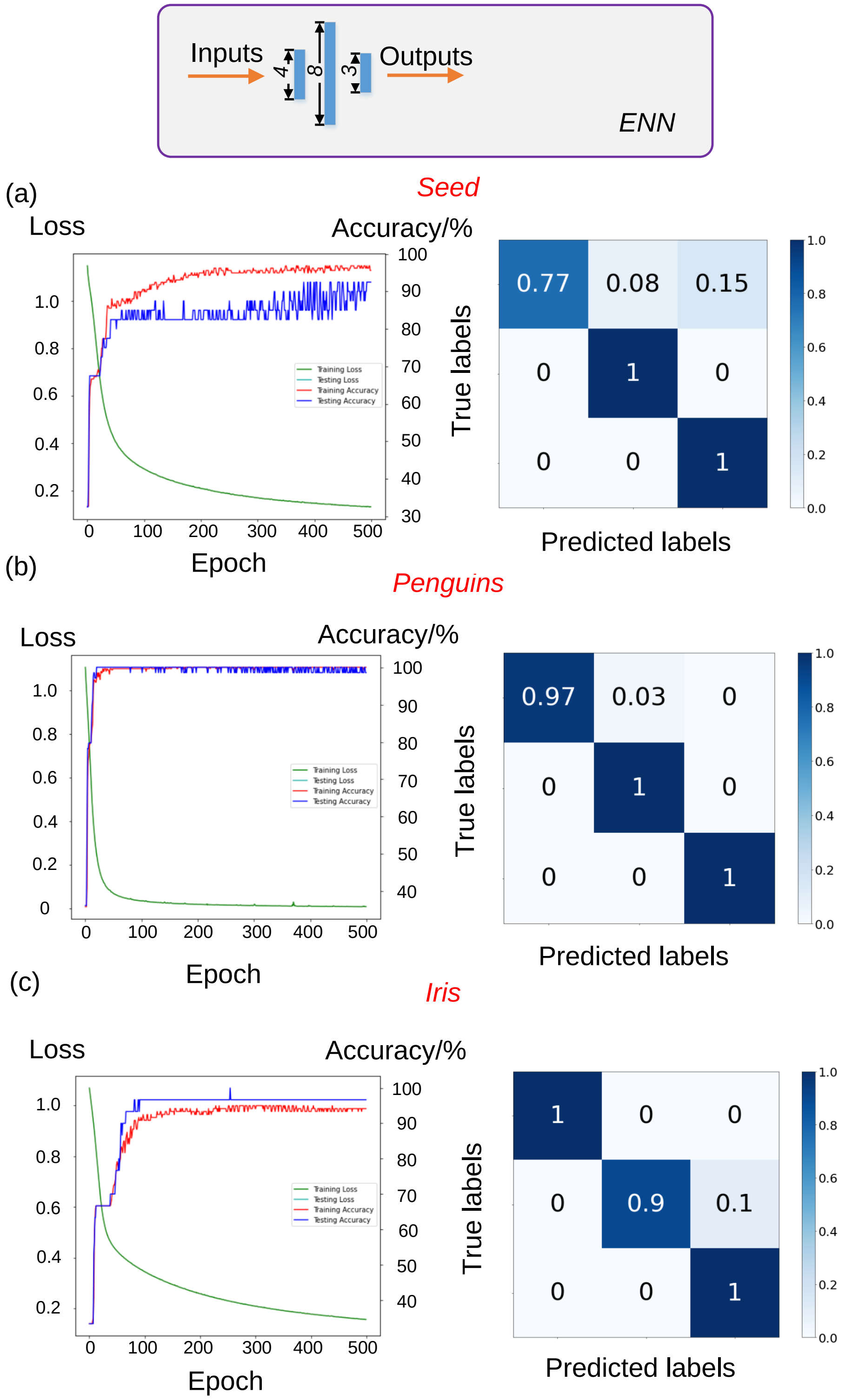

Figure S4: The accuracy and loss curves and confusion matrics of testing dataset for electrical net structure in (a) seed, (b) penguins, (c)iris dataset, respectively. The test accuracies for the 3 datasets are 92.5%, 98.6% and 96.7%, respectively.

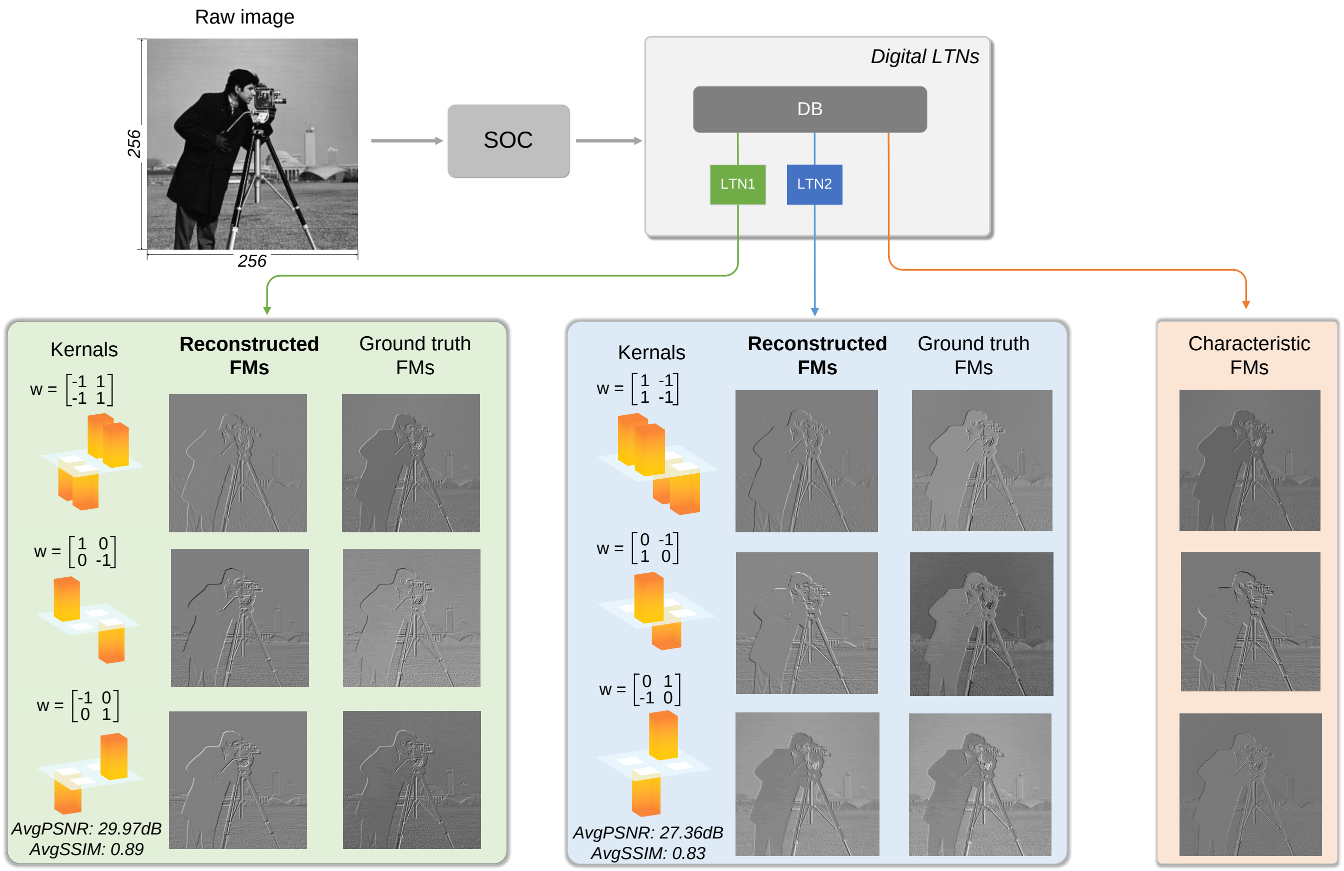

Figure S5: Another experimental image and the outputs of specific reconstructed convolution kernels and the characristic kernals of the SOC, compared with the ground truth from commercial 64-bit computer.

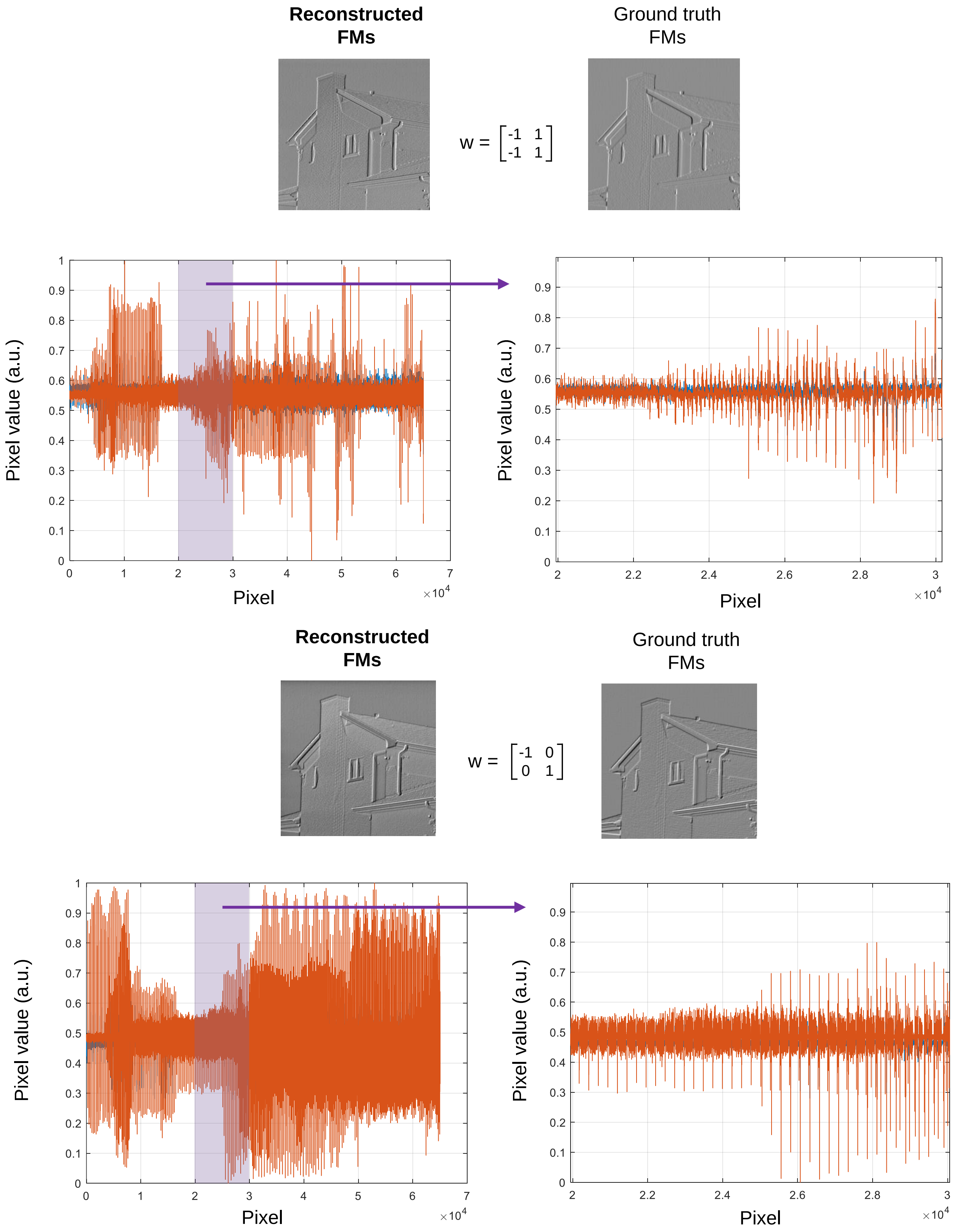

Figure S6: Comparasion of serialized pixels of the two produced feature map. Blue line, experimental results; Orange line, simulation results.

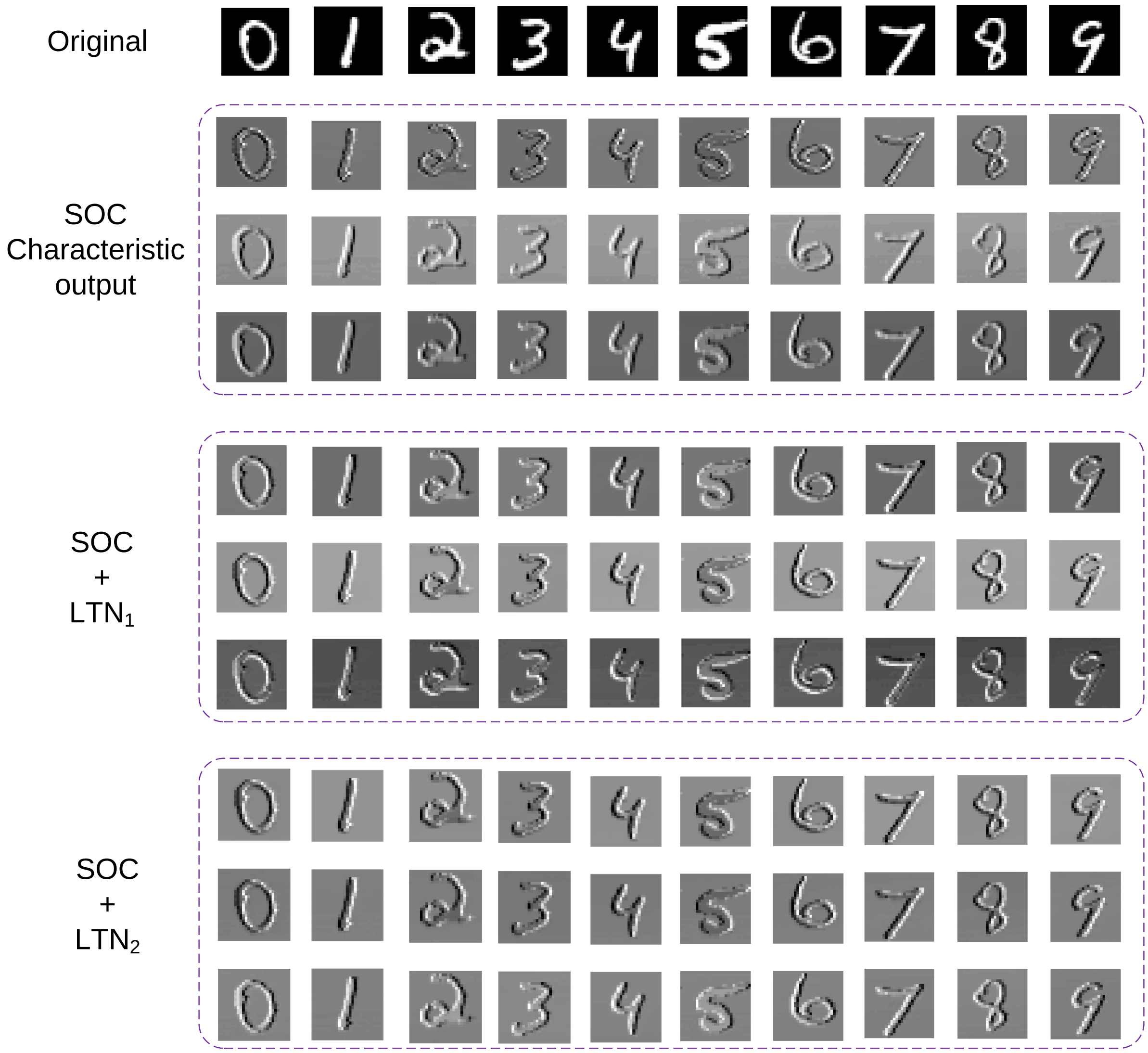

Figure S7: Experimental results of MNIST handwritten digits feature maps produced by SOC, SOC + LTN_1 and SOC + LTN_2, respectively.

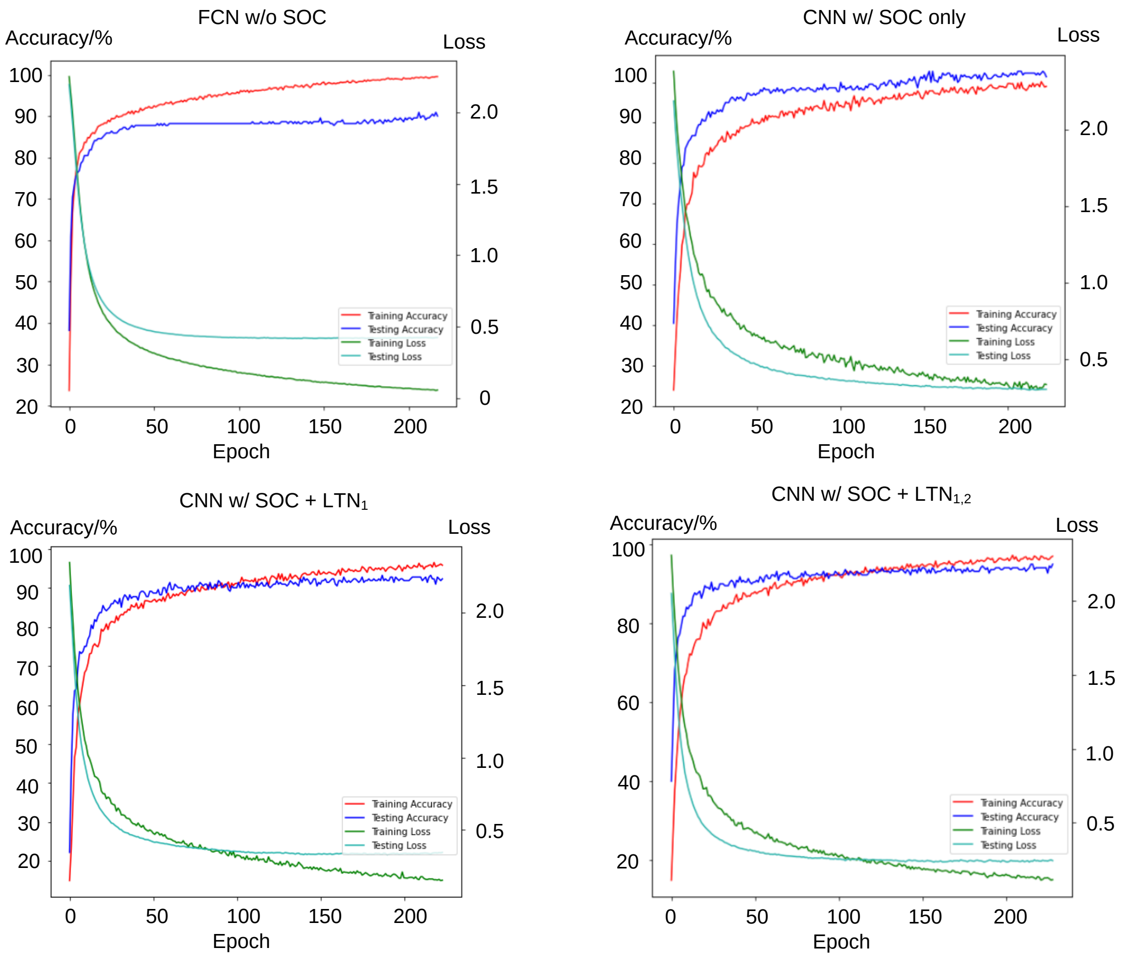

Figure S8: The accuracy and loss curves of MNIST handwritten digits dataset for four network structures. The traning dataset includes 2,000 images and the testing dataset includes 200 images.

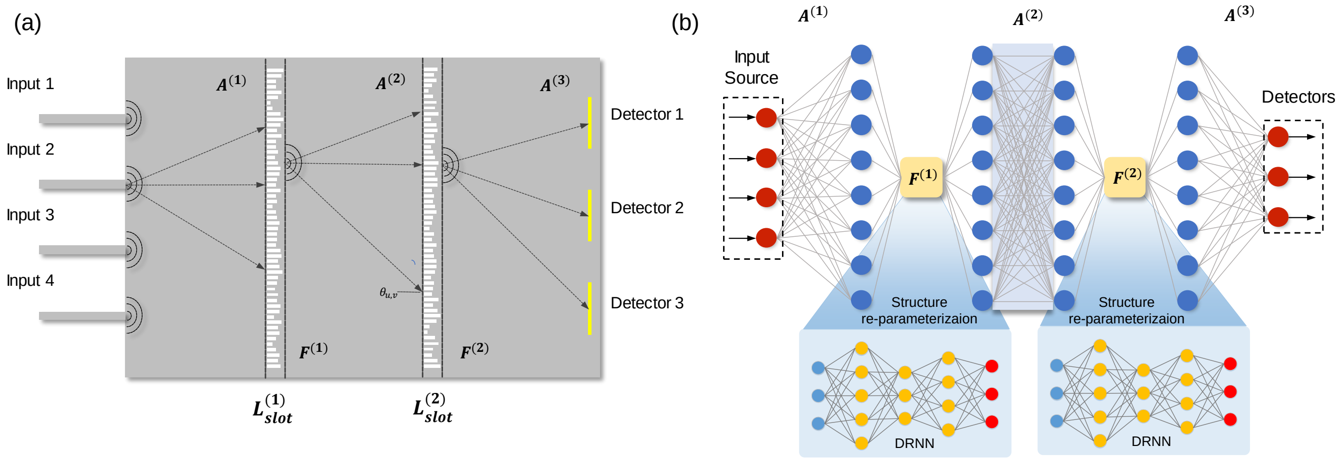

Figure S9: (a) Schematic of proposed SOC, each point on a given layer acting as a secondary source of a wave, the on-chip propagation in different area can be calculated by matrix A and function F respectively. (b) Network abstraction of forward propagation process. The martrix A can be represented by Huygens Fresnel principle and function F can be mapped to DRNN in Supplementary note 1.

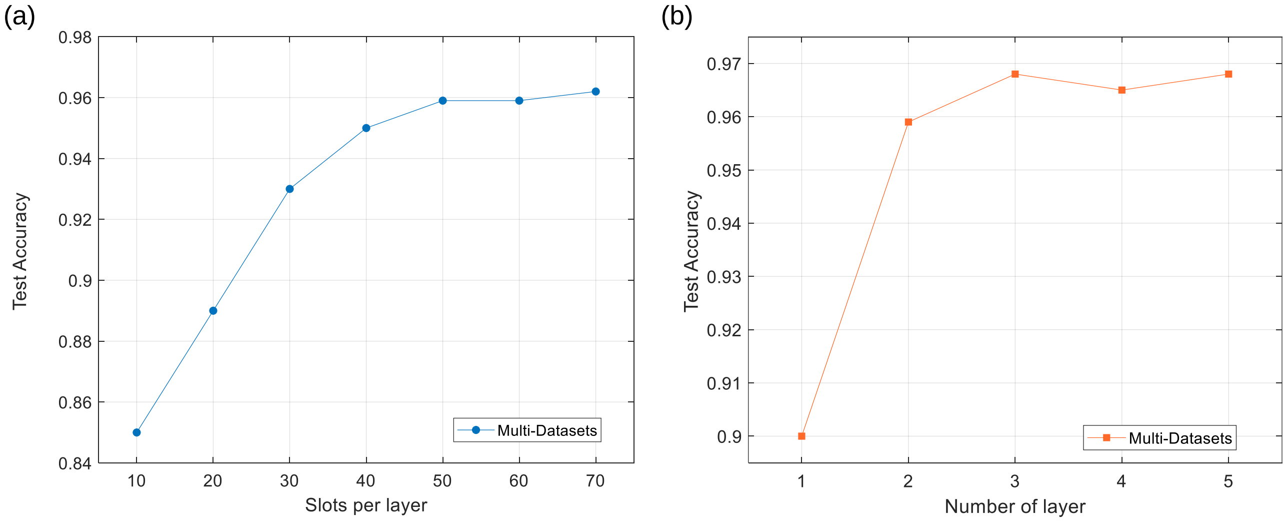

Figure S10: (a) Impact of the number of slots per layer on the classification accuracy. (b) Impact of the number of layers on the classification accuracy.

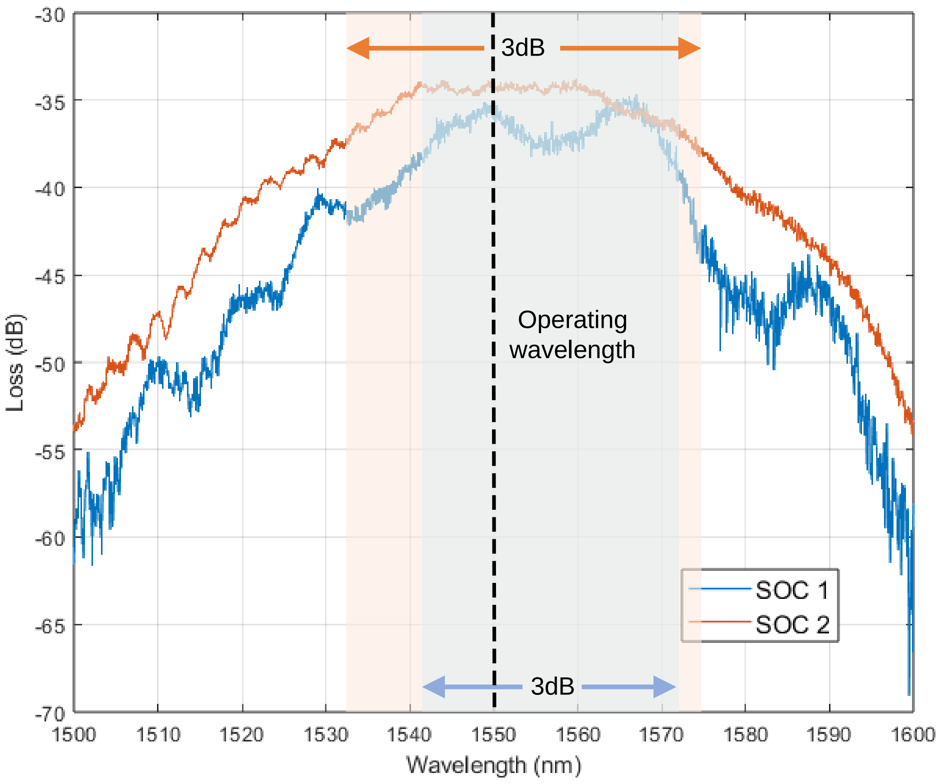

Figure S11: Experimental results of wavelength-scan of the two SOC chips.

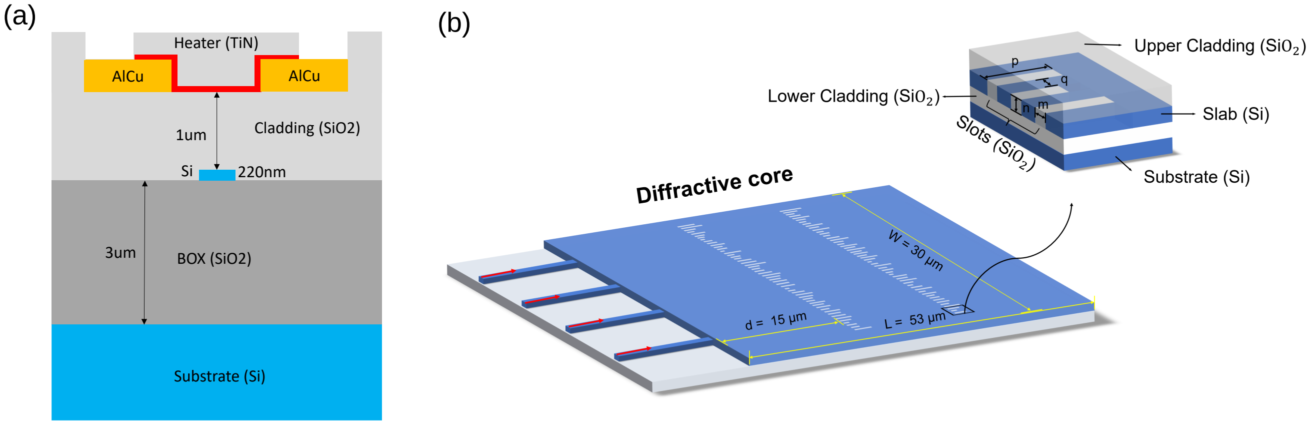

Figure S12: (a) The fabrication process details of our designed SOC chip. (b) Structure of the proposed SOC chip.

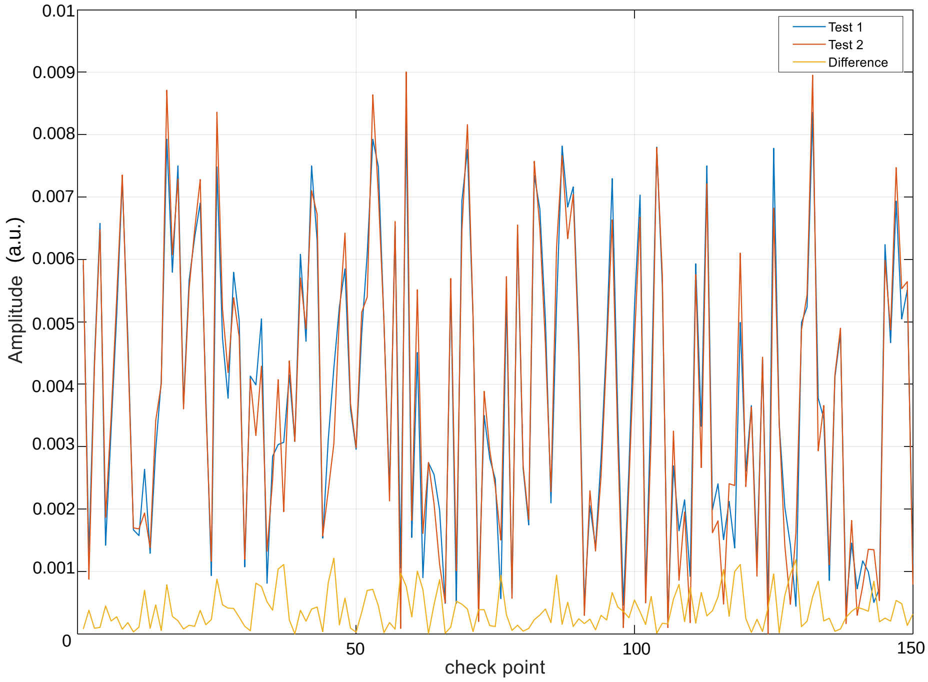

Figure S13: Comparison of test results for the same chip at two different times and under reconnection conditions.

References

[1] Tingzhao Fu, Yubin Zang, Yuyao Huang, Zhenmin Du, Honghao Huang, Chengyang Hu, Minghua Chen, Sigang Yang, and Hongwei Chen. Photonic machine learning with on-chip diffractive optics. *Nature Communications*, 14(1):70, 2023.

[2] Yuyao Huang, Tingzhao Fu, Honghao Huang, Sigang Yang, and Hongwei Chen. Sophisticated deep learning with on-chip optical diffractive tensor processing. *Photonics Research*, 11(6):1125–1138, 2023.

[3] Tingzhao Fu, Yubin Zang, Honghao Huang, Zhenmin Du, Chengyang Hu, Minghua Chen, Sigang Yang, and Hongwei Chen. On-chip photonic diffractive optical neural network based on a spatial domain electromagnetic propagation model. *Optics Express*, 29(20):31924–31940, 2021.

[4] Steven H Strogatz. Exploring complex networks. *nature*, 410(6825):268–276, 2001.

[5] Chiheb Trabelsi, Olexa Bilaniuk, Ying Zhang, Dmitriy Serdyuk, Sandeep Subramanian, Joao Felipe Santos, Soroush Mehri, Negar Rostamzadeh, Yoshua Bengio, and Christopher J Pal. Deep complex networks. *arXiv preprint arXiv:1705.09792*, 2017.

[6] Maxime W Matthès, Yaron Bromberg, Julien de Rosny, and Sébastien M Popoff. Learning and avoiding disorder in multimode fibers. *Physical Review X*, 11(2):021060, 2021.

[7] Wencan Liu, Tingzhao Fu, Yuyao Huang, Run Sun, Sigang Yang, and Hongwei Chen. C-donn: compact diffractive optical neural network with deep learning regression. *Optics Express*, 31(13):22127–22143, 2023.

[8] Yuyao Huang, Wencan Liu, Run Sun, Tingzhao Fu, Yaode Wang, Zheng Huang, Sigang Yang, and Hongwei Chen. Diffraction-driven parallel convolution processing with integrated photonics. *Laser & Photonics Reviews*, page 2400972, 2024.

[9] Zhihao Xu, Tiankuang Zhou, Muzhou Ma, ChenChen Deng, Qionghai Dai, and Lu Fang. Large-scale photonic chiplet taichi empowers 160-tops/w artificial general intelligence. *Science*, 384(6692):202–209, 2024.
